# GESearch: An Interactive GUI Tool for Identifying Gene Expression Signature

**DOI:** 10.1155/2015/853734

**Published:** 2015-06-25

**Authors:** Ning Ye, Hengfu Yin, Jingjing Liu, Xiaogang Dai, Tongming Yin

**Affiliations:** ^1^The Southern Modern Forestry Collaborative Innovation Center, Nanjing Forestry University, Nanjing 210037, China; ^2^College of Information Science and Technology, Nanjing Forestry University, Nanjing 210037, China; ^3^Research Institute of Subtropical Forestry, Chinese Academy of Forestry, Fuyang, Zhejiang 311400, China; ^4^Key Laboratory of Forest genetics and breeding, Chinese Academy of Forestry, Fuyang, Zhejiang 311400, China; ^5^College of Forest Resources and Environment, Nanjing Forestry University, Nanjing 210037, China

## Abstract

The huge amount of gene expression data generated by microarray and next-generation sequencing technologies present challenges to exploit their biological meanings. When searching for the coexpression genes, the data mining process is largely affected by selection of algorithms. Thus, it is highly desirable to provide multiple options of algorithms in the user-friendly analytical toolkit to explore the gene expression signatures. For this purpose, we developed GESearch, an interactive graphical user interface (GUI) toolkit, which is written in MATLAB and supports a variety of gene expression data files. This analytical toolkit provides four models, including the mean, the regression, the delegate, and the ensemble models, to identify the coexpression genes, and enables the users to filter data and to select gene expression patterns by browsing the display window or by importing knowledge-based genes. Subsequently, the utility of this analytical toolkit is demonstrated by analyzing two sets of real-life microarray datasets from cell-cycle experiments. Overall, we have developed an interactive GUI toolkit that allows for choosing multiple algorithms for analyzing the gene expression signatures.

## 1. Introduction

High-throughput gene expression technologies, such as microarray or RNA-seq, can rapidly generate expression profiles of a large number of transcripts at a time, which extract a snapshot of global expression at a certain cellular state of samples [1, 2]. Such technologies are powerful for exploring the genome-wide expression signatures of transcripts. However, the efficient and effective methods in the context of analyzing large expression datasets remain challenging.

To facilitate the data processing and mining, the original dataset is commonly needed to be transformed into a reduced-dimension matrix [3, 4]. Then, the unsupervised clustering algorithms, mainly including hierarchical clustering, *K*-means clustering, and self-organizing neural network (SOM), are widely employed to interpret the expression data [5–7]. Since formats of expression data vary greatly, clustering algorithms generally require to reformat the original data, which might cause loss of useful information [8, 9]. During clustering analysis, it is hard to determine the number of categories. A small number of categories tend to merge the unrelated groups into one category. On the contrary, a large number of categories lead to separating the related members into different categories [8, 9]. Therefore, clustering algorithms might be helpful in understanding the global profiles of gene expression but might not be suitable for identifying coexpression genes with defined expression signatures.

Identifying the gene expression signatures (or molecular signatures) is critical in different biological studies, such as studies on cancer [10, 11], on cell growth and differentiation [12], and on disease diagnose [13, 14]. The group of genes with similar expression profiles in response to internal or external factors could further be used to model biologically relevant networks, which were essential for better understanding the underlying molecular mechanisms [15, 16]. For instance, Pujana et al. constructed a network relevant to breast cancer by integrating the gene coexpression signatures with functional genomics data and by using prior known information of tumor suppressors [17]. More recently, Aijo et al. proposed methods based on nonparametric Gaussian process to characterize the time-course RNA-seq data and to determine temporally correlated genes during human T helper 17 cell differentiation [18]. More and more attentions have been paid to the extracting gene expression signatures, and there are several public databases, such as gene expression omnibus (GEO) [19, 20], molecular signatures database (MSigDB) [21, 22], and gene signatures database (GeneSigDB) [23], that provide useful platforms for analyzing the gene networks [19, 21–23]. There are also several public servers, such as CellMontage, CRCView, and FARO, that allow novel and content-based search for identifying gene expression signatures [24–26]. The efficient approaches for candidates identification and for functional analysis will allow developing new applicable biomarkers, as well as facilitating the understanding of biological processes. To date, several computing packages have been developed for identifying gene expression signatures based on similarity searching. For instance, Fujibuchi et al. defined the periodically expressed genes in different cell-cycle phases by using the sine and cosine vectors [27]; and Xiang et al. designed a method that queried data repositories based on gene expression patterns rather than textual annotations on gene expression omnibus (GEO) [28]. However, it is still challenging to identify meaningful groups of coexpressed genes in many biological scenarios. Different clustering algorithms might produce outputs of genes with distinctly different functions. So it is of great importance to provide a user-friendly platform allowing selecting and comparing of different algorithms and models for biologists. Also the capability of inputting priori knowledge is helpful for functional analysis of specific biological pathways.

In this study, we report an interactive GUI-based package written in Matlab, GEsearch, which can be easily used by click-and-pick. This package enables us to filter the input data, to import the prior knowledge for specific group of genes or expression profiles, and to choose different searching models. Another feature of this package is that the output of coexpression genes can be grouped based on expression abundance and be transferred or visualized for further analysis. We subsequently tested the feasibility of this package by using two independent gene expression datasets from cell-cycle experiments. The results showed that this package is not only efficient to find periodically expressed genes in different cell-cycle phases but also highly capable of predicting the downstream coexpressed genes regulated by a cell-cycle specific transcription factor. Taken together, this package is a useful analytical toolkit for dealing with large-scale gene expression datasets in functional genomic studies.

## 2. Methods and Implementation

**Figure 1:**
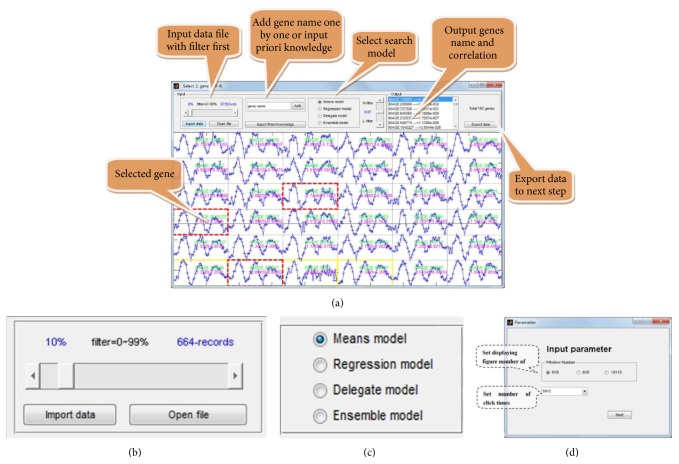
The input interface of GESearch. (a) The main window of GESearch, popping-up windows describe the utilities of software. Detailed manual is available at http://115.29.234.170/software/. (b) Panel for data filtering, (c) panel for selecting the searching models, and (d) panel for setting up parameters in GESearch package.

The GEsearch package was implemented in MATLAB. Users without access to the MATLAB need to use the MATLAB Runtime Compiler (MRC) for deploying the package. The GEsearch allows users to filter the input data and to select appropriate algorithms. Then, users can select the number of displaying genes within a window to browse the candidates (Figure 1(a)). Also, users can import expression data with prior knowledge to identify the coexpressed genes (Figure 1(a)). The package and user manual can be found on the webpage (http://115.29.234.170/software/).

### 2.1. Data Filtering and Preprocessing

Some gene expression profiles might not meet for the variation requirement (e.g., all-zero expression) and thus could not provide meaningful “signature” information. Such data would be eliminated in the analyzing process. To filter the input data, genes with small variations can be removed by selecting an appropriate threshold as described by the following formula (Figure 1(b)): $y_{i\sigma }=\sqrt{\left(1/N\right)\sum_{j=1}^{N}{\left(y_{ij}-\mu_{i}\right)}^{2}}$, where *y*_*ij*_ stands for the value of row *i*, column *j* in the gene expression dataset, *μ*_*i*_ represents the mean value of row *i*, *N* is the number of columns, and *y*_*iσ*_ is the standard deviation for data in row *i*. The range of values in row *i* is determined by formula: Δ_*i*_ = max⁡(*y*_*i*_) − min⁡(*y*_*i*_), where max⁡(*y*_*i*_) is the maximum value of data in row *i* and min⁡(*y*_*i*_) is the minimal value of data in row *i*. If Δ_*i*_ < *T*, the record will be removed in data analysis. In the established toolkit, a scrolling bar provides the threshold information for data filtering (Figure 1(b)), and *T* threshold for filtering is defined by the value from slider selection (Figure 1(b)).

### 2.2. Selections of Models

#### 2.2.1. The Mean Model

One has $\bar{y}=(\sum_{i=1}^{k}y_{i})/k$, where *k* stands for the number of selected genes, *y*_*i*_ is the expression level of gene *i*, and $\bar{y}$ represents the mean value of selected genes. The mean model is suitable for analyzing datasets with more uncertain prior knowledge, which calculates the average gene expression level. With this model, the similarity is strengthened by suppressing the Gaussian noise.

#### 2.2.2. The Regression Model

The regression model developed a scale-independent algorithm to fix the flaw of strong noise in the mean model. A nonlinear regression model is derived from the linear regression model through regression transformation listed as follows:

(1) yi=β0+β1x1+β2x2+⋯+βpxp+εi,εi∈N0,σ.

Construct *A* matrix:

(2) AX′X=N∑xi∑xi2⋯∑xip∑xi2∑xi3⋯∑xip+1⋯⋮∑xi2p,X1x1x12⋯x1p1x2x22⋯x2p⋮⋮⋮⋯⋮1xnxn2⋯xnp.

Calculate the coefficients of regression equation:

(3) $$\begin{array}{c} b=A^{-1}B={\left(\;X^{T}X\;\right)}^{-1}X^{\prime }Y. \end{array}$$

The regression model is suitable for datasets with gradient changes among sampling points, for example, time-series datasets.

#### 2.2.3. The Delegate Model

The delegate model selects a “delegate” based on a group of records. The delegate model can enhance the scale-independence ability. In many cases, the gene expression level varies greatly. This model is suitable for analyzing expression datasets with dramatic variation ranges.

Therefore *y* = max⁡(max⁡(*y*_*i*_) − min⁡(*y*_*i*_)), where max⁡(*y*_*i*_) and min⁡(*y*_*i*_) stand for maximal and minimal values of selected genes, respectively, and the one with maximal range value was chosen as delegate.

#### 2.2.4. The Ensemble Model

The ensemble model mixes the above three models to give the user a balanced solution for both accurate matching and general searching. The best optimization of this algorithm is to deliver a best-effort matching of all possible results. The ensemble model is developed for the flexible identification of candidates. For each record, this model extracts multiple signatures for similarity searching, which can provide more information for the final results. Comparing to the methods using single signature, ensemble model can be more efficient and accurate.

Therefore *y* = *UR*(*y*_*i*_)  *i* = 1 ⋯ *k*, where *k* stands for the number of selected genes, *U* stands for the total searched genes after removing redundancy, and *R*(*y*_*i*_) stands for the results by using gene *i*.

### 2.3. Multiple Rounds of Click-and-Pick Search

Users can start selecting genes of interest by randomly browsing the displayed genes. On the interface, options for the numbers of genes are provided. According to the size of the screen and the data, pop-up window allows user to pick an option from 6 × 6, 8 × 8, and 10 × 10 displaying genes (Figure 1(d)). At the same time, users are allowed to select the number of candidates for identifying coexpression genes (Figure 1(d)). If a subset of candidates is already identified, a pop-up window for importing priori knowledge will initialize the input of the designated gene group.

### 2.4. Characterization and Exportation of the Output Genes

**Figure 2:**
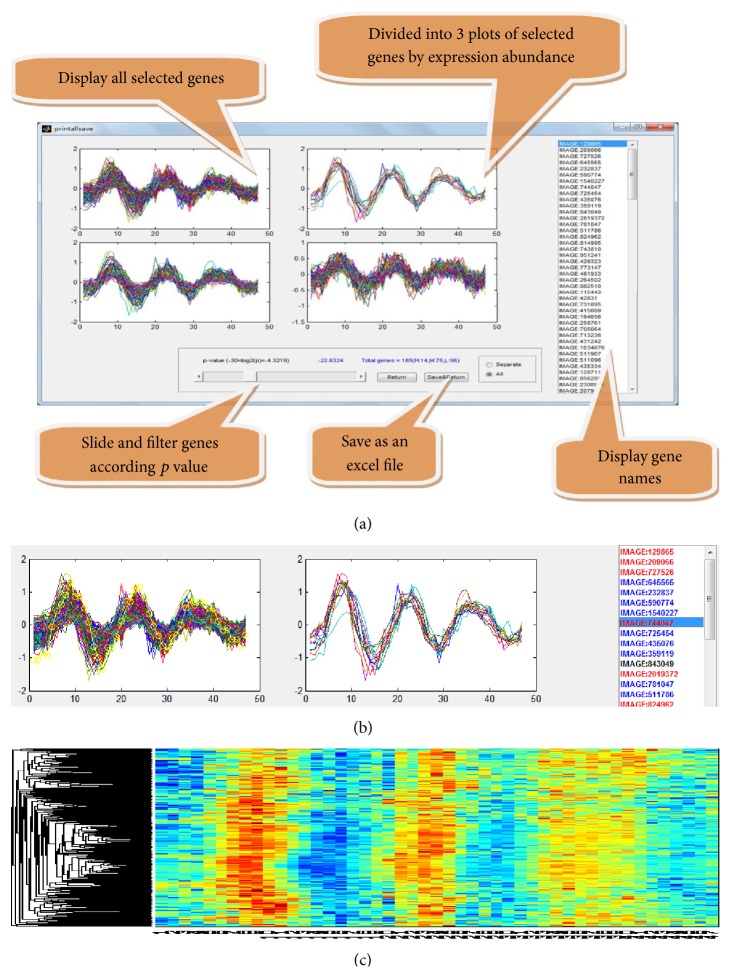
The main output interfaces of GESearch. (a) Popping-up windows display the utilities of output. (b) In “separate” model, results are split. (c) Heat map displays the final results.

There are four options of models, including the mean, the regression, the delegate, and the ensemble models, that can be selected for identifying the coexpressed genes (Figure 1(c)). After model selection, users can define the threshold of output data by scrolling the correlation *p* value. In the output panel, numbers of identified genes with correlation *p* values are shown (Figure 2(a)). After optimizing the output parameters, the results can be exported into a text file by selecting the “export data” button (Figure 2(a)). In the output window, the final results can be visualized into three separated panels according to expression abundance (Figure 2(a)). By selecting the “separate” button, the output data will be deposited in three files according to expression abundance. To better display the outputs, red color is used to highlight the highly expressed genes, and blue color is used to highlight the low expressed genes in the displaying window (Figure 2(b)). Furthermore, each output of genes can be easily visualized by the heat map (Figure 2(c)).

## 3. The Utility of GEsearch

**Figure 3:**
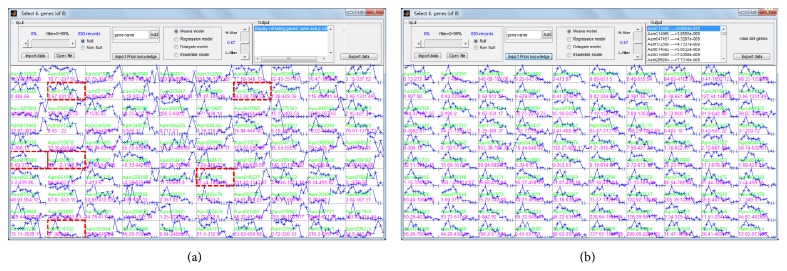
An example of random selecting gene profiles of interest. (a) Display the input data before searching. (b) Resulting panel after selection and search.

For initial exploration of gene expression dataset, a random selecting and searching approach can be efficient to identify genes of interest. For example, we developed an in-house RNA-seq dataset containing over 90,000 transcripts (derived from* de novo* assembly), with 8 time points. After randomly picked profiles of interest, GESearch found a group of 304 coexpressed genes with highly similar expression patterns (Figure 3). This approach is efficient and straightforward for small or moderate scale datasets in which gene expression signatures can be easily detected and visualized. To examine the feasibility of GESearch, we retrieved and analyzed two large gene expression datasets from human and yeast cell-cycle studies.

### 3.1. An Example of Using Priori Knowledge

**Figure 4:**
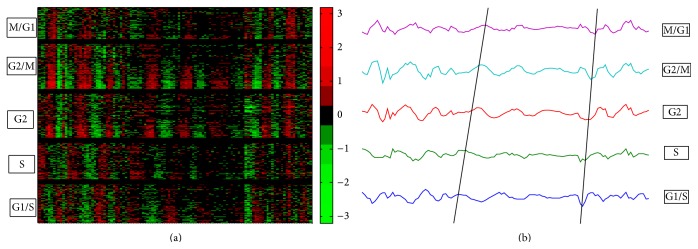
Results of using priori knowledge of cell-cycle marks in human Hela cell data. (a) Heat map displays the searching results. (b) Plotting the mean values of each group, in which black lines indicate the trends of peaks.

In this example, we selected the human Hela cell-cycle dataset to test the feasibilities of this package [27]. Previous studies have shown that there were specified marker genes representing phases of cell cycle, so we chose a subset of the dataset containing 118 time points and used 20 cell-cycle marker genes representing G1/S, S, G2, G2/M, and M/G1 as priori knowledge to search for the coexpressed periodic genes (http://genome-www.stanford.edu/Human-CellCycle/Hela/data/). This dataset also contained 42920 transcript IDs in which the periodic patterns were not easily seen. In this case, the use of prior known genes was essential for the identification of coexpression genes with defined expression signatures. By using the mean model and default filtering parameter (*p* value threshold < 0.67), the searching process identified five groups of genes which had very similar periodic expression patterns (Figure 4(a)). The final results were visualized by a heat map chart (Figure 4(a)). To further evaluate the accuracy of the output results, the mean values of each group were plotted together and the expected progression of cell-cycle phases was evident (Figure 4(b)). As a result, this example addressed a complex dataset by using priori knowledge, and the resulting groups of genes were ready for further functional analysis.

### 3.2. Identification of Downstream Targets of a Transcription Factor

**Figure 5:**
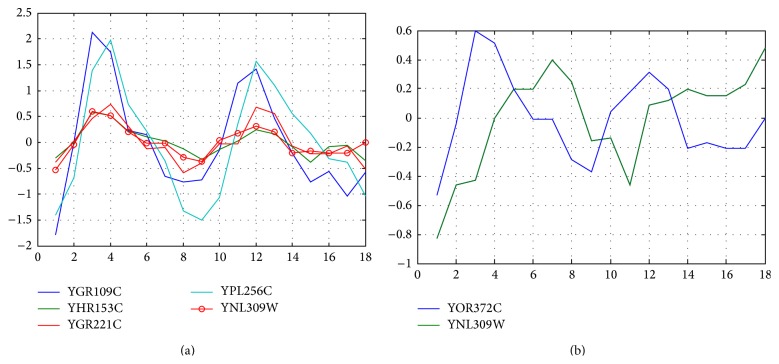
Coexpression analysis of yeast cell-cycle transcription factor YNL309W. (a) GESearch detected four cell-cycle target genes, including the known targets of YGR109C, YPL256C, and YGR221C, and the newly detected YHR153C. (b) The target gene of YOR372C was in shifted expression profiles, which was not detected by GESearch, but was identified by the time delay analysis.

Another useful application of coexpression analysis is to identify potential downstream targets for transcription factors, such as in yeast. The yeast cell-cycle data are from website (http://genome-www.stanford.edu/cellcycle/data/rawdata/) [29], which contains 6187 genes and 18 samples. We select yeast cell-cycle transcription factors MBF (YNL309W) as priori knowledge. Analyzed with GESearch, 74 genes were detected to have the similar expression pattern (results are not shown), among which YGR109C, YHR153C, YPL256C, and YGR221C had been proved to be the targets of MBF, and participating the regulation of cell cycle [30]. Contrast to this study, it was reported that there were 41 downstream targets of YNL309W (http://www.yeastract.com/), and four of them, including YGR109C, YOR372C, YGR221C, and YPL256C, were cell-cycle target genes (Figure 5(a)) [30, 31]. Compare these two studies; it was notable that YOR372C was not detected by GESearch. A detail check showed that the expression pattern of YOR372C shifted and peaked about 3-4 hours earlier than that of MBF (Figure 5(b)). This observation showed that YOR372C did not coexpress with MBF and explained why it was not detected by GESearch. It was also noteworthy that our search engine detected a new cell-cycle gene (YHRL153C) in the coexpression group of MBF (Figure 5(a)), which encoded the G1 cycling activating G1 to S phase transition, and its expression level was regulated by MBF complex [31, 32]. This example confirmed that the GESearch package was efficient in finding coexpression genes as well as in predicting the target genes of transcription factor.

## 4. Conclusions and Discussion

The design of this package follows the logic that gene expression profiles contain biologically relevant signatures; gene expression signatures are predictable and complement for understanding biological processes. And these notions have been extensively investigated by studies on cancer. For example, gene expression analysis of microarray data has played pivotal roles in breast cancer classification, prognostication, and prediction [33, 34]. It was noteworthy that gene expression signatures based predictions were severely affected by the measuring methods and selected datasets [34]. To obtain reliable and repeatable results from gene signatures extraction, multiple choices and comparisons of algorithms are essential.

GESearch provided a comprehensive meaning for finding coexpression genes set of interest, and its multiple choices of searching models allowed rapid identification, regardless of dependent variables. Moreover, this analytical platform is user friendly. As shown previously, the output datasets from cell-cycle experiments were ready for visualization (Figure 2). With the availability of functional information, output datasets could be transformed to be adopted for the functional enrichment tools [35]. GESearch was not only limited to coexpression analysis [36] but also useful for identifying the downstream candidates. Although the time delay analysis was successful for target prediction, the resolution of sampling affected the outcomes of expression signatures due to the specific interaction of transcription factors [37]. It needs further investigations on the defined co- and shift-expressed genes to find the true downstream targets of a transcription factor.

In conclusion, GESearch provides an interactive platform for exploiting the large-scale gene expression datasets. Its choice of multiple models is more users friendly for identifying the coexpressed genes, and its options for data output and visualization provide an efficient way to integrate data, which can help the further analyses.
